# The Effects of Nitrogen Addition on the Uptake and Allocation of Macro- and Micronutrients in *Bothriochloa ischaemum* on Loess Plateau in China

**DOI:** 10.3389/fpls.2017.01476

**Published:** 2017-08-24

**Authors:** Zemin Ai, Guoliang Wang, Chutao Liang, Hongfei Liu, Jiaoyang Zhang, Sha Xue, Guobin Liu

**Affiliations:** ^1^State Key Laboratory of Soil Erosion and Dryland Farming on the Loess Plateau, Institute of Soil and Water Conservation, Northwest A&F University Yangling, China; ^2^Institute of Soil and Water Conservation, Chinese Academy of Sciences and Ministry of Water Resources Yangling, China; ^3^Research Center of Soil and Water Conservation and Ecological Environment, University of Chinese Academy of Sciences Beijing, China; ^4^College of Forestry, Northwest A&F University Yangling, China

**Keywords:** forage plant, artificial management, concentration, storage, ratio of above- to belowground tissues, soil total nitrogen

## Abstract

The effects of nitrogen (N) addition on the macro- and micronutrient concentrations, storage, and allocation of *Bothriochloa ischaemum* (L.) Keng, a native forage plant on the Loess Plateau in China remain unclear. We studied the effects of N addition at 0 (CK), 2.5 (N1), 5.0 (N2), and 10.0 (N3) g N m^-2^ y^-1^. N addition significantly decreased the available copper (Cu), zinc (Zn), and total Cu concentration, but significantly increased the available iron concentration in the soil. Cu, manganese (Mn), and sodium (Na) concentrations in aboveground tissues and potassium (K), magnesium, and Zn concentrations in belowground tissues significantly increased with N addition. Calcium (Ca) concentrations in belowground tissues decreased significantly. The ratios of above- to belowground Ca, Cu, Zn, and Mn significantly increased with N addition. The maximum ratios appeared at N2 for Cu, Zn, and Mn. The aboveground, belowground, and total biomass storage of studied nutrients significantly changed with N addition, and most attained maximum values under N2 treatment. The storage ratios of above- to belowground Cu, Zn, Mn, and Na attained maximum values at N2. We conclude that N addition significantly, but differentially influence the macro- and micronutrient concentrations and storage in *B. ischaemum*. *B. ischaemum* allocated and accumulated increased macro- and micronutrients to its aboveground tissues and exhibited high total storage when the amount of N addition reached 5 g N m^-2^ y^-1^.

## Introduction

Minerals are necessary for the maintenance of certain physicochemical processes and are essential for plants. These inorganic substances are used by plants in many ways. Important minerals such as potassium (K), calcium (Ca), magnesium (Mg), and sodium (Na) are required in higher amounts by plants and are considered macronutrients. Macronutrients play a key role in plant growth and development and are incorporated into the structure of plants. For instance, K maintains balance in plant water potential, transports photosynthate, controls the opening and closing of stomata, and activates enzyme activity (Havlin et al., 2005). Mg plays an important role in chlorophyll molecule, adenosine diphosphate, and adenosine triphosphate and is closely related to anion malate and citrate (Taiz and Zeiger, 2010). Ca plays essential roles in regulating physiological processes, such as regulating membrane permeability and influencing the division of plant cells, which are closely related to plant growth and stress (Mclaughlin and Wimmer, 1999). Na is a beneficial element in some plants and is especially important to C4 plants that thrive in arid and semiarid regions. Under these growing conditions, Na is necessary for regulating the opening and closing of stomata (Havlin et al., 2005).

Plants require some minerals in small amounts, such as copper (Cu), iron (Fe), manganese (Mn), and zinc (Zn). These minerals are called micronutrients, which are involved in a series of enzyme formation and metabolic processes in plants; micronutrient deficiency can lead to diseases and affects the normal growth of plants (Khoshgoftarmanesh et al., 2010). For example, Cu plays an important role in the various Cu protein processes of plants for photosynthesis, respiration, and lignification; Cu can affect the carbohydrate and N metabolism of plants, such as through the enzyme involved in the electron transport chain of photosystem I (Mortvedt, 1991). Fe plays an essential role in oxidation–reduction reactions as an electron transport element and is an important component of cytochromes and haemes; Fe maintains the structure and function of chloroplast; Fe in chloroplasts accounts for approximately 80% of the total Fe in leaves (Terry and Abadía, 1986). Mn is an essential component of Mn-containing superoxide dismutase, which protects the plant’s singlet oxygen and hydrogen peroxide by converting them into H_2_O_2_ and water (Mukhopadhyay and Sharma, 1991). Zn is involved in the synthesis of substrates and enzymes and is involved in the regulation of gene transcription (Havlin et al., 2005; García-Bañuelos et al., 2014). Thus, given the essential role of macro- and micronutrients in plant growth and physiological metabolism, the concentrations of these minerals should be examined when comprehensively evaluating plant growth. This information can provide a scientific reference for the artificial management of vegetative restoration.

N is one of the most common limiting nutrients in plant growth and development (Vitousek and Howarth, 1991). Increased N input will obviously affect photosynthetic rates, growth rates, and plant productivity in most ecosystems (Xia and Wan, 2008; Granath et al., 2009; Bai et al., 2010). Experiments involving N addition are widely used as a strategy for agricultural management to alleviate ecosystemic N limitations, which could potentially influence macro- and micronutrient concentrations in plant soils (Malhi et al., 1998; Brennan, 2005; Sinkhorn, 2007; Treseder, 2008; Fang et al., 2012; Tian et al., 2015, 2016; Wang et al., 2017). A series of research were conducted on the effects of N fertilizer on macro- and micronutrients concentrations on plants and soils. Previous studies showed that N fertilizer increased the concentrations of K, Ca, Mg, Na, Zn, and Mn in aboveground tissues (Hamilton et al., 1998; Fang et al., 2012; Tian et al., 2016). However, other studies found that Ca, Na, Zn, and Mn concentrations in aboveground tissues decreased across an N gradient (Hamilton et al., 1998; Brennan, 2005; Sinkhorn, 2007; Fang et al., 2012). The available concentrations of micronutrients in the soil also exhibit different levels under N application. Most studies indicated that N addition results in significant increases in the availabilities of micronutrients, such as the available concentrations of Cu, Mn, and Fe in soils (Malhi et al., 1998; Tian et al., 2015; Tian et al., 2016; Wang et al., 2017), which significantly increased after N addition. However, Malhi et al. (1998) indicated that the concentrations of Cu and Zn in soils significantly decreased under higher treatments of N. These discrepant results show that changes in macro- and micronutrient concentrations in plants and soils vary widely probably because of differences in the year of fertilization, plant species, and soil properties (Zhang and Shan, 2000; Cheng et al., 2010; Fang et al., 2012; Simic, 2015; Tian et al., 2016). Moreover, only a few studies examined the allocation of macro- and micronutrient concentrations in plants and the influence of the main soil nutrients on the macro- and micronutrient concentrations in soils.

Atmospheric N deposition greatly influences the grassland ecosystem of Loess Plateau in China (Liu et al., 2011). At present, the N deposition rate in this region is 2.2 g N m^-2^ y^-1^; this level is expected to increase (Wei et al., 2010; Liu et al., 2013). Furthermore, the soil N level on Loess Plateau is low (Zhu et al., 1983); therefore, N may be one of the most limiting nutrients in this region (Vitousek and Howarth, 1991; Yang et al., 2011). N application may be one of the most important methods used for alleviating N deficiency in the grassland ecosystems of Loess Plateau of China. *Bothriochloa ischaemum* (L.) Keng, a perennial C4 grass with a culm cluster, is mainly distributed in warm temperate zones. This species has a specially developed root system that forms a root network. This species propagates rapidly, has strong resistance to trampling, tolerates drought, can adapt to harsh environments, and prevents soil erosion. *B. ischaemum* is an excellent native forage species with strong capability to regenerate on Loess Plateau. This characteristic is mainly attributed to its capability to adapt to local conditions and the palatability and forage quality of its aboveground tissues (Xu et al., 2011). However, the effects of N addition on macro- and micronutrient concentrations, storage, and their allocation to *B. ischaemum* tissues and concentrations in the soils on Loess Plateau remain unclear. Thus, appropriate concentration of N fertilizer should be selected and changes in macro- and micronutrient concentrations in plants and soils should be examined. Existing studies should also determine whether the concentrations of macro- and micronutrients in the aboveground forage tissues meet animal requirements after N application.

We experimentally added N at rates of 0, 2.5, 5.0, and 10.0 g N m^-2^ y^-1^ in soil tanks to study the effects of N addition on the macro- and micronutrient concentrations of *B. ischaemum* plants and surrounding soils. We tested the following hypotheses: (1) N addition would significantly influence the available concentrations of micronutrients, but not the total concentrations of macro- and micronutrients in the soil. (2) N addition would significantly but differentially influence the macro- and micronutrient concentrations and storage in the above- and belowground tissues of plants, as well the concentration and storage ratios from above- to belowground. (3) Soil N would be closely related to the macro- and micronutrient concentrations in soil, and different N components would have different effects.

## Materials and Methods

### Plant Materials

The seeds of *B. ischaemum* were obtained in the late autumn of 2012 from the *B. ischaemum* community in the experimental fields at the Ansai Research Station (ARS) of the Chinese Academy of Sciences (36°51′30″N, 109°19′23″E; 1068–1309 m a.s.l.). ARS is located in the center of the semiarid, hilly gully region of the Loess Plateau in Northwestern China. The seeds were sun-dried under natural, dry conditions and stored in sealed plastic bags at a laboratory in Yangling, Shaanxi Province. The germination rates of the seeds were above 90% when germinated on moist filter paper in Petri dishes at 25°C before the experiment (Xu et al., 2011).

### Growth Conditions

The experiment was conducted under a rainout shelter at the State Key Laboratory of Soil Erosion and Dryland Farming on Loess Plateau in Yangling (34°12′N, 108°7′E; 530 m a.s.l.). The mean annual temperature is 12.9°C, with average temperatures of -2°C and 26.7°C in January and July, respectively. The mean annual precipitation is 637.6 mm. The loessial soils used for this experiment were collected from an arable field at the ARS. The soils were sandy loam with 55% porosity; a bulk density of 1.2 g cm^-3^; an organic matter content of 1.3 g kg^-1^; and total N (TN) and phosphorus (TP) concentrations of 0.19 and 0.27 g kg^-1^, respectively. The gravimetric water content of the soils at field capacity and the wilting point were 20.0 and 4.0%, respectively. The soils were air-dried and passed through a 2 mm mesh.

### Experimental Design

The experiment began in June 2013. The soils were added to soil tanks (200.0 cm × 100.0 cm × 50.0 cm; length × width × height) in 10 cm layers to a total depth of 40 cm (Wu et al., 2008) with slopes of 15° gradients (Chen et al., 2007). The following five treatments were conducted: without *B. ischaemum* or added N (BL), *B. ischaemum* without added N (CK), *B. ischaemum* with 2.5 g N m^-2^ y^-1^ (N1), *B. ischaemum* with 5 g N m^-2^ y^-1^ (N2), and *B. ischaemum* with 10 g N m^-2^ y^-1^ (N3). Each treatment was replicated thrice to obtain a total of 15 soil tanks. The soils in the soil tanks were well watered before sowing to ensure seedling establishment. *B. ischaemum* seeds were sown into holes at a density of 10 cm × 10 cm. Soil moisture content was maintained at above 80% of field capacity during seedling establishment. Excess grass plants were manually removed to restrict plants to one per hole of the same size based on field studies at the ARS of *B. ischaemum* growth density. N was applied in 2013 and 2014 as urea, which is a widely used N fertilizer in China (Tian et al., 2016). The urea used in the experiment was produced for agricultural use according to GB 2440-2001 of the People’s Republic of China. This urea does not contain K, Ca, Mg, Na, Cu, Fe, Mn, and Zn. In August 2013, N was applied as a one-time application of urea solution in a 1:l ratio with deionized water. In 2014, the same total amount was applied over four equal applications in May, June, July, and August. For N fertilization started being applied after the plants reached a certain size, and therefore the chosen rate of N application was done in one time on the first year, but divided over four times on the second year (corresponding to different growth stages). Both BL and CK treatments received the same volume of water.

### Sampling

All plant and soil samples were obtained at the end of August 2014. The aboveground plant tissues in all soil tanks were harvested from the soil surface using scissors. The belowground tissues and soil samples (in the 0–20 cm soil layer, obtained with a soil core sampler with 2.5 cm diameter) were collected from six 20 cm × 20 cm quadrats in each soil tank after the aboveground tissues were removed. The belowground tissues were washed carefully with water over a 60-mesh screen until the roots were separated from the soil. The above- and belowground tissues and soil samples in each soil tank were combined to form a sample. Finally, 24 plant samples and 15 soil samples were obtained. Plant samples with constant weight were dried at 65°C in an oven and then weighed, ground through a 1 mm sieve, and then stored for chemical analysis. All soil samples were air-dried. After the roots, stones, and debris were removed, the soil samples were homogenized and sieved to 0.25, 1, and 2 mm prior to analysis. All soil samples were then stored for chemical analysis. The above- and belowground biomasses of *B. ischaemum* were analyzed in another paper (Ai et al., 2017) and are shown in **Table 1**.

**Table 1:** The above- and belowground biomass of *B. ischaemum* in each treatment.

		Treatment			
		CK	N1	N2	N3
Biomass (g m^-2^)	Aboveground	253.3^d^	536.8^c^	861.5^a^	825.8^b^
	Belowground	123.6^d^	268.9^b^	304.2^a^	240.7^c^


*Different letters above the bars on the same line indicate a significant difference at P = 0.05*.

### Laboratory Analysis

The available Cu, Zn, Mn, and Fe were obtained by extracting 10 g of dry soil (sieved to 2 mm) with 20 mL of diethylenetriaminepentaacetic acid (DTPA) solution (0.005 M DTPA + 0.01 M CaCl_2_ + 0.1 M triethanolamine, pH 7.3) (Lindsay and Norvell, 1978). After 2 h of continuous shaking at room temperature, the soil suspension was centrifuged and filtered through a 0.45 mm membrane. Cu, Zn, Mn, and Fe in the extract were analyzed with an atomic absorption spectrometer (GBC932AA). The total soil K, Ca, Na, Mg, Cu, Zn, Mn, and Fe were analyzed by digesting 0.1 g of soil with HClO_4_–HNO_3_–HF (Agemian and Chau, 1976; Tessier et al., 1979). The digested solution was washed in a flask and deionized water was added to a fixed volume. The total plant K, Ca, Na, Mg, Cu, Zn, Mn, and Fe were digested with HClO_4_–HNO_3_ (Sparks et al., 1996). All digested solutions were analyzed with an atomic absorption spectrometer (GBC932AA).

Soil organic carbon (SOC) concentration was determined using the H_2_SO_4_–K_2_Cr_2_O_7_ oxidation method, and the soil TN concentration was measured using the Kjeldahl method (Bremner and Mulvaney, 1982). The soil TP concentration was determined colorimetrically after digestion with H_2_SO_4_ and HClO_4_, and the soil available phosphorus (SAP) was measured with the Olsen method (Olsen and Sommers, 1982). Soil ammonium-N (AN) and nitrate-N (NN) were extracted with 2 M KCl and quantified colorimetrically on an ALPKEM AutoAnalyzer (OI Analytical, College Station, TX, United States). The soil water-soluble SOC (W-SOC), water-soluble AN (W-AN), water-soluble NN (W-NN), and water-soluble TN (W-TN) were extracted using deionized water. The soil water-soluble organic-N (W-SON) concentration was calculated as the W-TN concentration (W-NN concentration + W-AN concentration) (Jones and Willett, 2006). Soil pH was determined in a water-to-soil ratio of 2.5:1 with an automatic acid-based titrator (Metrohm 702, Swiss).

### Data Analysis

Aboveground/belowground biomass macro- and micronutrient storage = aboveground/belowground biomass × aboveground/belowground biomass macro- and micronutrient concentration, total biomass macro- and micronutrient storage = aboveground biomass macro- and micronutrient storage + belowground biomass macro- and micronutrient storage. Data from the treatments were analyzed using one-way analysis of variance (ANOVA) followed by Duncan’s test. Correlation analysis was conducted based on Pearson’s correlation coefficients. All ANOVAs and correlation analyses were tested for significance at *P <* 0.05 using SPSS 20.0 (SPSS Inc., Chicago, IL, United States), and structural equation models (SEMs) were analyzed using the SPSS Amos expansion pack. Redundancy analysis (RDA), a direct gradient analysis, was performed using CANOCO 5.0 (Biometris, Wageningen, Netherlands). The graphs were plotted using SigmaPlot 12.5 (Systat Software, San Jose, CA, United States).

## Results

### Available Concentrations of Micronutrients in Soil

**FIGURE 1:**
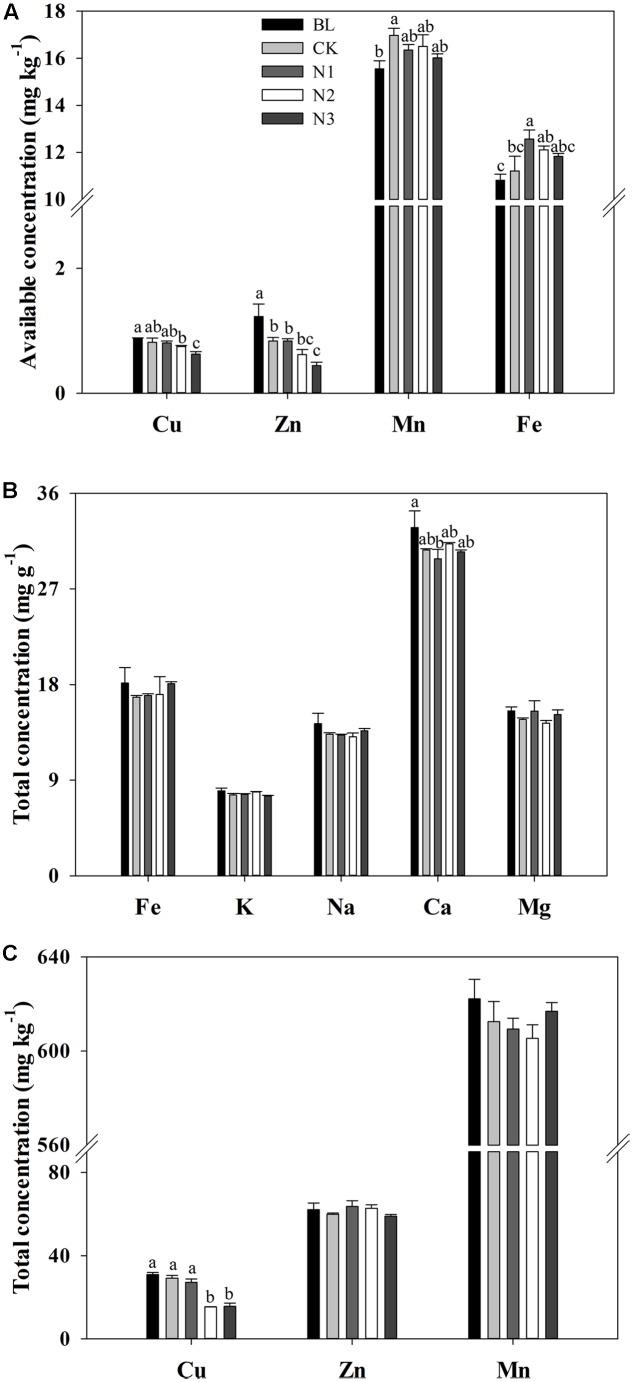
Effects of N addition on macro- and micronutrient concentrations in the soils of *B. ischaemum*. **(A)** The available concentrations of Cu, Zn, Mn, and Fe in soils of *B. ischaemum*. **(B)** The total concentrations of Fe, K, Na, Ca, and Mg in soils of *B. ischaemum*. **(C)** The total concentrations of Cu, Zn, and Mn in soils of *B. ischaemum*. Error bars are SE (*n* = 3). Different letters above bars indicate a significant difference at *P* = 0.05.

The available concentrations of Cu, Zn, and Fe significantly changed with the N addition, and the available Cu and Zn had maximum values in the BL treatment and significantly decreased with increasing N addition (**Figure 1A**). The minimum value of available Fe concentration appeared in the BL treatment, which increased significantly at N1 (which was not significant at N2 and N3) in comparison with CK. With Fe, the minimum concentration of available Mn occurred in the BL treatment, but it did not significantly change with the addition of N (**Figure 1A**).

### Total Concentrations of Macro- and Micronutrients in Soil

The total concentrations of Fe, K, Na, Mg, Mn, and Zn in the soil did not significantly change with the addition of N, whereas the total concentration of Ca decreased significantly with N addition (**Figures 1B,C**). No significant difference was observed in total Cu concentration among BL, CK, and N1 treatments. Total Cu concentration significantly decreased in the N2 and N3 treatment levels. Total Ca concentration in the BL treatment did not exhibit significant difference from that in the CK treatments, but decreased significantly in N1 treatment.

### Macro- and Micronutrient Concentrations in the Above- and Belowground Biomass and their Ratios

**FIGURE 2:**
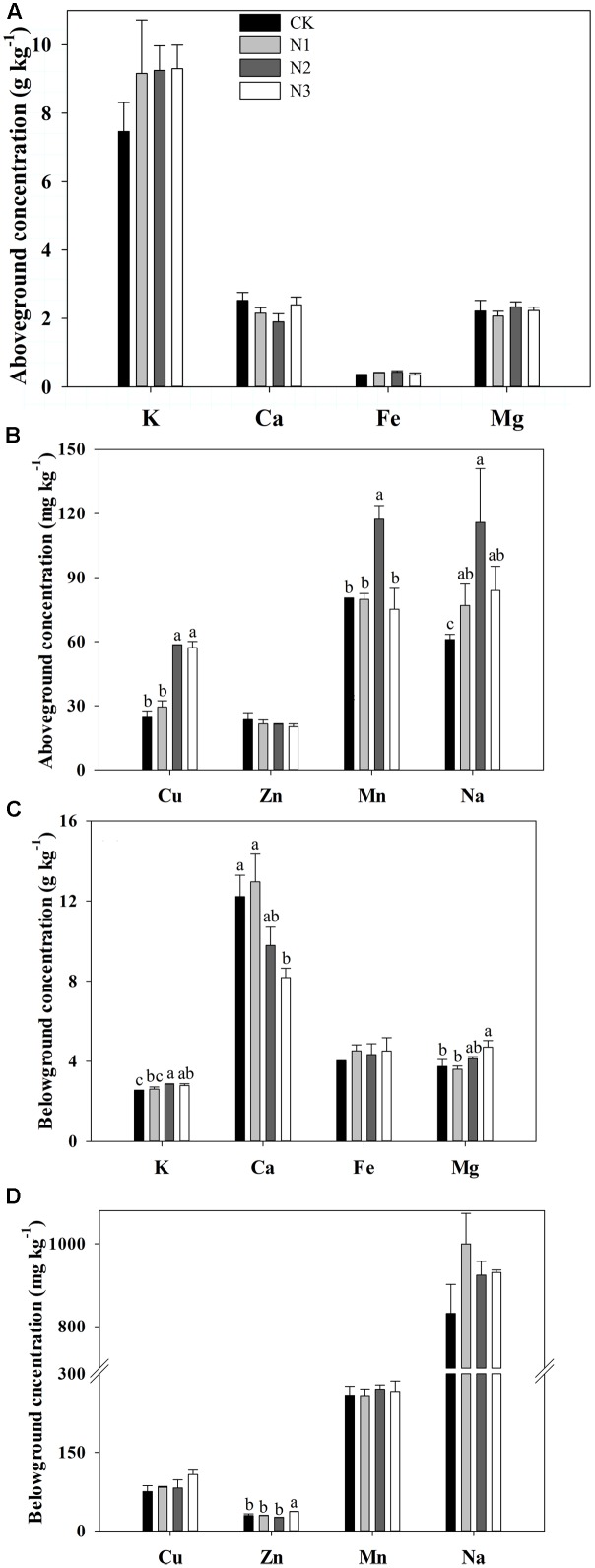
Effects of N addition on plant biomass macro- and micronutrient concentrations in *B. ischaemum*. **(A)** The concentrations of K, Ca, Fe, and Mg in the aboveground biomass of *B. ischaemum*. **(B)** The concentrations of Cu, Zn, Mn, and Na in the aboveground biomass of *B. ischaemum*. **(C)** The concentrations of K, Ca, Fe, and Mg in the belowground biomass of *B. ischaemum*. **(D)** The concentrations of Cu, Zn, Mn, and Na in the belowground biomass of *B. ischaemum*. Error bars represent SE (*n* = 3). Different letters above bars indicate a significant difference at *P* = 0.05.

N addition significantly affected the Na, Cu, and Mn concentrations in the aboveground biomass, but the Fe, K, Ca, Mg, and Zn concentrations in the aboveground biomass were not affected (**Figures 2A,B**). Na, Cu, and Mn concentrations significantly increased with increasing N addition and reached a maximum value in the N2 treatment. N2 concentrations significantly increased by 90, 138, and 46% respectively, compared with CK. Cu and Mn concentrations significantly increased until the fertilizer was at the N2 level. Mn concentration significantly decreased in the N3 treatment compared to N2, whereas Cu concentration did not change under N3 treatment.

K, Ca, Mg, and Zn concentrations in the belowground biomass varied significantly with N addition, but Na, Cu, Mn, and Fe concentrations did not change (**Figures 2C,D**). The K concentration increased significantly at N2 and N3 but not at N1 compared with CK. Zn and Mg concentrations significantly increased at high treatment levels, whereas Ca concentration significantly decreased with increasing addition of N.

The concentration ratios of above- to belowground Ca, Cu, Zn, and Mn varied and were significantly affected by N addition, but the concentration ratios of above- to belowground K, Na, Mg, and Fe were not affected (**Table 2**). The concentration ratios of above- to belowground Cu and Mn at N2 significantly increased by 134 and 39% in comparison with CK. The concentration ratio of above- to belowground Mn at N3 significantly decreased by 56% compared with N2. However, the increase was slower at higher amounts of added N. The concentration ratio of above- to belowground Ca was maximal in N3 compared with N1, and significantly increased by 75%.

**Table 2:** The concentration and storage ratios of above- to belowground biomass macro- and micronutrient of *B. ischaemum* in each treatment.

		Treatment			
		CK	N1	N2	N3
The concentration ratio of above- to belowground biomass	Cu	0.33^b^	0.35^b^	0.78^a^	0.54^ab^
	Zn	0.81^a^	0.74^ab^	0.84^a^	0.55^b^
	Mn	0.31^b^	0.31^b^	0.44^a^	0.28^b^
	Na	0.08^a^	0.08^a^	0.13^a^	0.09^a^
	Fe	0.08^a^	0.09^a^	0.10^a^	0.08^a^
	K	2.94^a^	3.48^a^	3.25^a^	3.35^a^
	Ca	0.21^ab^	0.17^b^	0.20^ab^	0.30^a^
	Mg	0.62^a^	0.58^a^	0.56^a^	0.48^a^
The storage ratio of above- to belowground biomass	Cu	0.69^b^	0.70^b^	2.22^a^	1.84^a^
	Zn	1.67^b^	1.47^b^	2.39^a^	1.87^ab^
	Mn	0.65^c^	0.62^c^	1.24^a^	0.97^b^
	Na	0.15^b^	0.15^b^	0.36^a^	0.31^ab^
	Fe	0.17^a^	0.18^a^	0.30^a^	0.27^a^
	K	6.10^b^	6.94^b^	9.18^ab^	11.52^a^
	Ca	0.43^b^	0.35^b^	0.56^b^	1.02^a^
	Mg	1.26^a^	1.16^a^	1.60^a^	1.63^a^


*Different letters above the bars in the same line indicate a significant difference at P = 0.05*.

### Macro- and Micronutrient Storage in Above- and Belowground Biomass and their Ratios

**FIGURE 3:**
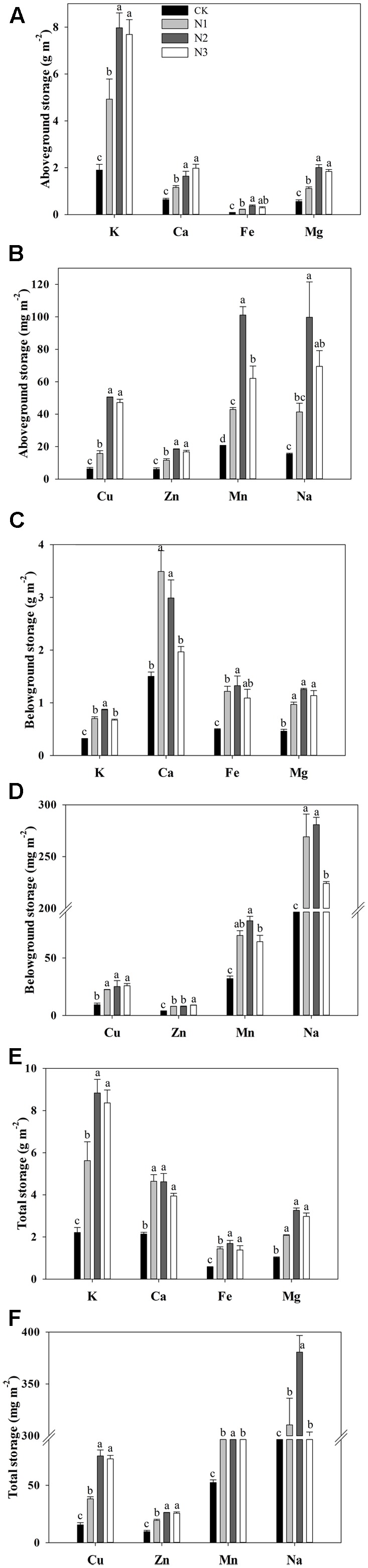
Effects of N addition on plant biomass macro- and micronutrient storage of *B. ischaemum*. **(A)** The storage of K, Ca, Fe, and Mg in the aboveground biomass of *B. ischaemum*. **(B)** The storage of Cu, Zn, Mn, and Na in the aboveground biomass of *B. ischaemum*. **(C)** The storage of K, Ca, Fe, and Mg in the belowground biomass of *B. ischaemum*. **(D)** The storage of Cu, Zn, Mn, and Na in the belowground biomass of *B. ischaemum*. **(E)** The total of K, Ca, Fe, and Mg in the biomass of *B. ischaemum*. **(F)** The total storage of Cu, Zn, Mn, and Na in the biomass of *B. ischaemum*. Error bars represent SE (*n* = 3). Different letters above the bars indicate a significant difference at *P* = 0.05.

The storage of K, Ca, Fe, Mg, Cu, Zn, Mn, and Na in the aboveground biomass responded significantly to the N addition (**Figures 3A,B**). The storage of macro- and micronutrients significantly increased with increasing N addition, and most of them reached a maximum value at N2, with the exception of Ca, wherein the maximum value was observed in N3. Compared with CK, the storage of K, Ca, Fe, Mg, Cu, Zn, Mn, and Na at N2 treatment significantly increased by 320, 157, 337, 259, 705, 208, 394, and 545%, respectively. However, aboveground Mn storage decreased significantly at the highest treatment levels.

The storage of macro- and micronutrients in the belowground biomass varied significantly with N addition across treatments (**Figures 3C,D**). The storage of Cu and Fe increased significantly at N1 compared with CK and remained similar across high treatment levels. The storage of Ca, Mn, and Na increased significantly at N1 compared with CK. The storage of Ca and Na decreased significantly at N3 compared with N2. The storage of K increased significantly with N addition until N2 and decreased significantly at N3 compared with N2. The storage of Zn and Mg significantly increased with increasing N addition.

The total storage of macro- and micronutrients in *B. ischaemum* biomass varied significantly with N addition across treatments (**Figures 3E,F**). The total storage of K, Mg, Cu, Zn, Mn, and Na increased significantly under low N addition, but did not change under the highest N addition, except for Mn and Na, which decreased significantly under the highest N addition. The total storage of K, Mg, Cu, Zn, Mn, and Na attained maximum values in N2 at 8.84 g m^-2^, 3.26 g m^-2^, 75.61 mg m^-2^, 26.21 mg m^-2^, 183.45 mg m^-2^, and 380.64 mg m^-2^, respectively. The total storage of Ca and Fe increased significantly under the lowest N addition and did not change with increasing N addition. The total Fe storage achieved a maximum value at N2, reaching 1.69 g m^-2^, whereas the maximum value for Ca was 4.64 g m^-2^ at N1.

The macro- and micronutrient storage ratios of above- to belowground levels were significantly altered by N addition, but Fe and Mg did not change (**Table 2**). The storage ratios of above- to belowground Cu, Zn, Mn, and Na did not increase significantly at N1, only at N2 compared to CK, and the storage ratios of above- to belowground Mn decreased at N3 compared with N2. The storage ratios of above- to belowground K and Ca vary similarly to Cu, Zn, Mn, and Na and increased significantly at N3 in compared to CK.

### Effects of Main Soil Nutrient Factors on Soil Macro- and Micronutrient Concentrations

**FIGURE 4:**
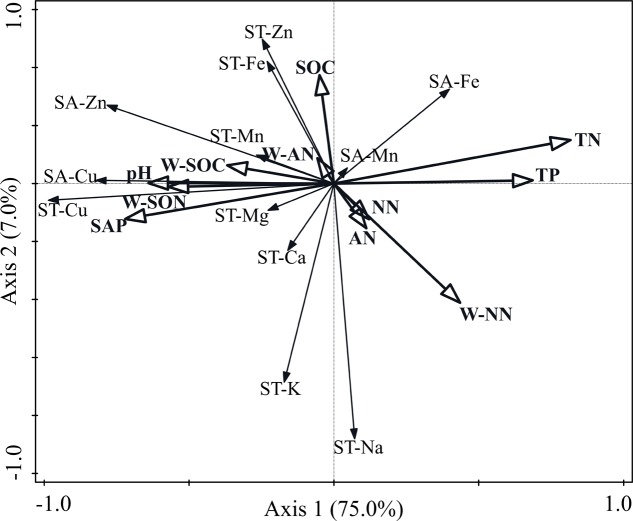
Bi-dimensional graph for the redundancy analysis indicating the relationships between soil macro- and micronutrient concentrations and soil nutrient variables. SA-Cu, soil available-Cu concentration; SA-Zn, soil available-Zn concentration; SA-Mn, soil available-Mg concentration; SA-Fe, soil available-Fe concentration; ST-Cu, soil total-Cu concentration; ST-Zn, soil total-Zn concentration; ST-Mn, soil total-Mn concentration; ST-Fe, soil total-Fe concentration; ST-K, soil total-K concentration; ST-Na, soil total-Na concentration; ST-Ca, soil total-Ca concentration; ST-Mg, soil total-Mg concentration; SOC, soil organic-carbon concentration; TN, total-nitrogen concentration; TP, soil total-phosphorus concentration; SAP, soil available-phosphorus concentration; NN, soil nitrate-nitrogen concentration; AN, soil ammonium-nitrogen concentration; W-SOC, soil water-soluble organic-carbon concentration; W-NN, soil water-soluble nitrate-nitrogen concentration; W-AN, soil water-soluble ammonium-nitrogen concentration; W-SON, soil water-soluble organic-nitrogen concentration.

Constrained redundancy analysis (RDA) indicated that soil nutrient factors affected the macro- and micronutrient concentrations of the soil (**Figure 4**). The total variation was 2.23, and the explanatory variables accounted for 94.2%. The first two axes explained 82.0% of the total variance, wherein 75.0% was attributed on the first axis and 7.0% on the second axis. Soil TN concentration was the most significant variable among the 11 soil factors and explained 50.4% (*P* = 0.002) of the total variance. The SAP and W-SON concentrations were the next most significant environmental variables and explained 9.2% (*P* = 0.036) and 6.9% (*P* = 0.044) of the total variance, respectively.

### Path Analysis

**FIGURE 5:**
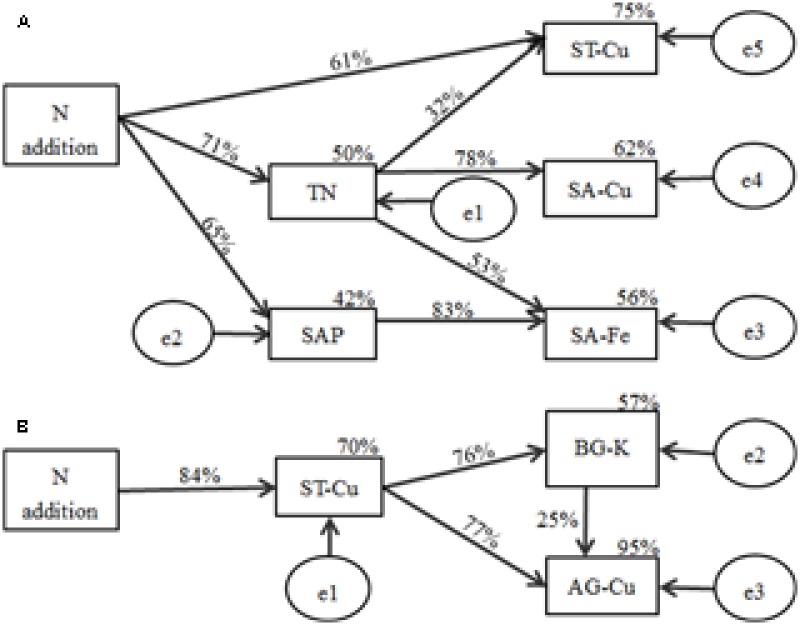
Structural equation model results for the effects of N addition on macro- and micronutrient concentrations in **(A)** soil and **(B)** plant biomass of *B. ischaemum*. Numbers on arrows represent the standardized path coefficients (equivalent to correlation coefficients). Circles indicate error terms (e1–e5). Percentages close to endogenous variable.

According to the indices, the final SEM model adequately fitted the data to describe the effects of soil nutrient factors on soil macro- and micronutrient concentrations (*x*^2^= 5.254, *P* = 0.730, RMSEA *P <* 0.001; standardized path coefficients are shown in **Figure 5A**). The final model accounted for 50% of the variation in TN, with 42% of variation in SAP, 56% of variation in SA-Fe, 75% of variation in ST-Cu, and 62% of variation in SA-Cu. N addition exhibited a negative relationship with SAP (*P* < 0.01) and ST-Cu (*P* < 0.01) and a positive relationship with TN (*P* < 0.001). TN exhibited a negative relationship with SA-Fe (*P* < 0.05) and SA-Cu (*P* < 0.001). SAP (*P* < 0.001) exhibited a negative relationship with SA-Fe (*P* < 0.001).

A good model fit was found by all indices (*x*^2^= 1.071, *P* = 0.585, RMSEA *P <* 0.001; standardized path coefficients are shown in **Figure 5B**). This finding indicates the relationship between soil macro- and micronutrient concentrations and the macro- and micronutrient concentrations in plant tissues. The model was able to explain 70% of the variation in ST-Cu, 57% in BG-K, and 95% in AG-Cu. N addition had a strong negative relationship with ST-Cu (*P* < 0.001). ST-Cu also had a strong negative relationship with BG-K (*P* < 0.001) and AG-Cu (*P* < 0.001). BG-K had a positive relationship with AG-Cu (*P* < 0.05).

### Correlation Analysis

The correlation analysis showed that the storage of macro- and micronutrients had a significantly positive relationship with biomass and Cu concentration in the aboveground tissue of *B. ischaemum* (**Table 3**). Na concentration in the aboveground tissue also showed a good relationship with the storage of macro- and micronutrients in the aboveground tissue.

**Table 3:** Correlation analysis between concentrations and storage in the plant aboveground tissues of *B. ischaemum*.

		Concentrations								
		AGB	AG-Cu	AG-Zn	AG-Mn	AG-Na	AG-Fe	AG-K	AG-Ca	AG-Mg
Storage	AGS-Cu	0.965^∗∗^	0.988^∗∗^	-0.269	0.479	0.569	0.278	0.366	-0.314	0.178
	AGS-Zn	0.970^∗∗^	0.924^∗∗^	-0.123	0.501	0.631^∗^	0.353	0.513	-0.400	0.099
	AGS-Mn	0.876^∗∗^	0.856^∗∗^	-0.181	0.801^∗∗^	0.670^∗^	0.451	0.252	-0.455	0.288
	AGS-Na	0.834^∗∗^	0.783^∗∗^	-0.189	0.561	0.930^∗∗^	0.040	0.479	-0.536	0.152
	AGS-Fe	0.906^∗∗^	0.855^∗∗^	-0.271	0.554	0.376	0.666^∗^	0.286	-0.382	0.114
	AGS-K	0.945^∗∗^	0.867^∗∗^	-0.225	0.284	0.663^∗^	0.188	0.684^∗^	-0.472	-0.042
	AGS-Ga	0.884^∗^	0.849^∗^	-0.320	0.219	0.320	0.227	0.271	0.053	0.164
	AGS-Mg	0.970^∗∗^	0.906^∗∗^	-0.347	0.525	0.580^∗^	0.279	0.274	-0.345	0.344


*^∗^P < 0.05; ^∗∗^P < 0.01. AGB, aboveground biomass; AG-Cu, aboveground Cu concentration; AG-Zn, aboveground Zn concentration; AG-Mn, aboveground Mn concentration; AG-Fe, aboveground Fe concentration; AG-K, aboveground K concentration; AG-Na, aboveground Na concentration; AG-Ca, aboveground Ca concentration; AG-Mg, aboveground Mg concentration; AGS-Cu, storage of aboveground Cu concentration; AGS-Zn, storage of aboveground Zn concentration; AGS-Mn, storage of aboveground Mn concentration; AGS-Fe, storage of aboveground Fe concentration; AGS-K, storage of aboveground K concentration; AGS-Na, storage of aboveground Na concentration; AGS-Ca, storage of aboveground Ca concentration; AGS-Mg, storage of aboveground Mg concentration*.

Consistent with the aboveground tissue, the relationship between storage of macro- and micronutrients and biomass was also significantly positive in the belowground tissue of *B. ischaemum* (**Table 4**). However, Cu and Na concentrations in the belowground tissue had no relationship with the storage of macro- and micronutrients in the belowground tissue, and the K concentrations in the belowground tissue had a good relationship with the storage of macro- and micronutrients in the belowground tissue.

**Table 4:** Correlation analysis between concentrations and storage in the belowground part of *B. ischaemum*.

		Concentrations								
		BGB	BG-Cu	BG-Zn	BG-Mn	BG-Na	BG-Fe	BG-K	BG-Ca	BG-Mg
Storage	BGS-Cu	0.808^∗∗^	0.723^∗∗^	0.220	0.277	0.439	0.369	0.578^∗^	-0.325	0.362
	BGS-Zn	0.841^∗∗^	0.543	0.367	0.239	0.534	0.382	0.564	-0.401	0.481
	BGS-Mn	0.968^∗∗^	0.253	-0.133	0.404	0.459	0.444	0.607^∗^	-0.272	0.242
	BGS-Na	0.964^∗∗^	0.210	-0.129	0.156	0.693^∗^	0.284	0.604^∗^	-0.122	0.194
	BGS-Fe	0.885^∗∗^	0.275	-0.067	0.430	0.439	0.695^∗^	0.623^∗^	-0.136	0.178
	BGS-K	0.986^∗∗^	0.222	-0.172	0.207	0.493	0.323	0.712^∗∗^	-0.295	0.249
	BGS-Ga	0.754^∗∗^	0.024	-0.320	0.049	0.610^∗^	0.339	0.264	0.431	-0.188
	BGS-Mg	0.914^∗∗^	0.344	0.073	0.268	0.483	0.281	0.708^∗∗^	-0.445	0.554


*^∗^P < 0.05; ^∗∗^P < 0.01. BGB, belowground biomass; BG-Cu, belowground Cu concentration; BG-Zn, belowground Zn concentration; BG-Mn, belowground Mn concentration; BG-Fe, belowground Fe concentration; BG-K, belowground K concentration; BG-Na, belowground Na concentration; BG-Ca, belowground Ca concentration; BG-Mg, belowground Mg concentration; BGS-Cu, storage of belowground Cu concentration; BGS-Zn, storage of belowground Zn concentration; BGS-Mn, storage of belowground Mn concentration; BGS-Fe, storage of belowground Fe concentration; BGS-K, storage of belowground k concentration; BGS-Na, storage of belowground Na concentration; BGS-Ca, storage of belowground Ca concentration; BGS-Mg, storage of belowground Mg concentration*.

## Discussion

### Effects of N Addition on Soil Available Concentrations of Micronutrients

In our study, the available concentration of Mn in the soil did not exhibit a significant difference among N additions, which was inconsistent with our hypothesis. However, previous studies found that N addition caused large increases in available Mn concentrations (Li et al., 2007; Tian et al., 2015, 2016; Wang et al., 2017). The soil available concentrations of Cu, Fe, and Zn were significantly affected by N addition, which supported our hypothesis. The available concentrations of Cu and Zn significantly decreased at higher treatment levels, which was consistent with the findings of other studies (Malhi et al., 1998). However, some studies found that the available concentration of Cu significantly increased under long-term N addition (Tian et al., 2015; Wang et al., 2017). This difference may be attributed to year of fertilization, plant species, and soil (Cheng et al., 2010; Fang et al., 2012; Simic, 2015). The available concentrations of Cu may decrease because N addition can accentuate Cu deficiency when organic N compounds form within the plant in response to N addition (Bussler, 1981). Cu is an important component of enzymes that affect N metabolism in plants (Mills and Jones, 1996). Thus, N addition may increase the level of enzymes in plants that influence N metabolism, thereby forcing the plant to absorb a large amount of Cu from the soil and cause the concentration of Cu to decrease. However, soil TN would increase due to N addition. Therefore, soil TN and soil Cu concentrations would have a strong negative correlation, which is consistent with our RDA and path analysis (**Figures 4, 5**). The available concentration of Fe significantly increased with N addition, which is consistent with other studies (Malhi et al., 1998; Tian et al., 2015, 2016). High SAP can reduce Fe solubility by immobilizing Fe (Mandal and Haldar, 1980). Thus, the relationship between SAP and available Fe would be negative, which is supported by our RDA and path analysis (**Figures 4, 5**). When N addition promoted the uptake of SAP, a high level of Fe would be released, thereby increasing the available Fe in soil. The RDA and path analysis also showed that soil TN had a good relationship with available concentrations of micronutrients in the soil (**Figures 4, 5**). This finding is consistent with our hypothesis that soil N is closely related to the micronutrients in soil and different N components have different effects.

### Effects of N Addition on the Total Concentrations of Macro- and Micronutrients in Soil

Most of the total macro- and micronutrient concentrations in soil did not significantly change under N addition, except Cu. The results generally support our hypothesis, which is consistent with other studies (Li et al., 2007; Duan and Chang, 2015). However, Li et al. (2007) found that the total soil Cu concentration did not exhibit significant change under N fertilization, which was different from our results. As mentioned above, the relationship between soil Cu and soil TN was significantly negative. Therefore, N addition would significantly reduce total Cu concentration. The result supported by the RDA and path analyses (**Figures 4, 5A**) was consistent with our hypothesis. However, other studies found that the total soil Cu concentration was positively correlated with the soil TN concentration (Wu et al., 2010). The difference in soil properties may be one of the main factors (Cheng et al., 2010; Simic, 2015).

### Effects of N Addition on the Concentrations of Macro- and Micronutrients in Above- and Belowground Biomass

N addition significantly affected the aboveground biomass Cu, Mn, and Na concentrations, but it did not affect those in the belowground tissues. K, Ca, Mg, and Zn concentrations in the belowground biomass varied significantly with N addition, but they did not affect those in the aboveground tissues (**Figures 2A–D**). These results are consistent with our hypothesis and reflect how N addition alters the macro- and micronutrient uptake in above- and belowground tissues of plants. One study found that K, Ca, Mg, and Zn concentrations in the aboveground tissues increased with the level of N supply (Hamilton et al., 1998). In our study, the same trend was observed for K, Mg, and Zn concentrations in the belowground tissues, and N addition had no significant influence on K, Ca, Mg, and Zn concentrations in the aboveground tissues. Moreover, studies found that Ca and Zn concentrations decreased across the N gradient in the aboveground tissues (Brennan, 2005; Sinkhorn, 2007). In our study, Ca concentration significantly decreased in the belowground part, which is consistent with the findings of other research (Fang et al., 2012). Mn concentration in our study increased significantly under lower N addition, but decreased significantly under higher treatment levels, which is inconsistent with the findings of other studies (Hamilton et al., 1998; Tian et al., 2016). Previous studies found that N addition had a significant effect on Na concentrations in belowground tissues of different plant species (Fang et al., 2012), which is inconsistent with our study. However, the aboveground Na concentration significantly increased with increasing N addition. Therefore, the macro- and micronutrient concentrations in plant biomass were different among different plant parts, species, and regions under N addition. These differences may be attributed to differences in plant species and soil properties (Zhang and Shan, 2000; Fang et al., 2012; Simic, 2015).

Path analyses showed that the aboveground biomass Cu concentration had a significant negative correlation with the soil total Cu (**Figure 5B**). However, plant Cu mainly derives from soil Cu, and the absorption of Cu by plant belowground tissues would be given priority over aboveground tissues (Brennan, 2005). Thus, a negative correlation may exist between aboveground biomass Cu and soil Cu concentrations. A significant negative correlation between belowground biomass K and soil total Cu concentrations was found during path analyses (**Figure 5B**). The adsorption of micronutrients by roots is controlled by the concentration of other elements in the soil (Taiz and Zeiger, 2010). Thus, the absorption of K by the belowground tissues was possibly affected by the concentration of soil Cu.

Certain micronutrients are essential in supporting the health, growth, and reproduction of animals. Animals obtain a high portion of required minerals from forage plants, and adequate quantities of macro- and micronutrients in *B. ischaemum* are necessary to meet the nutrient requirements of animals. Therefore, changes in macro- and micronutrients are important for grazing livestock after N addition. Aboveground biomass Cu, Mn, and Na concentrations were changed significantly under N addition. According to Healy et al. (2016) a concentration range of Cu of 10–20 mg kg^-1^ is necessary to meet the nutrient requirements of animals. In our study, the concentration of Cu in the aboveground tissues was close to the concentration of the animal requirement at N1 (29.4 mg kg^-1^, **Figure 2B**). The aboveground concentration of Cu at N2 (58.6 mg kg^-1^, **Figure 2B**) was higher than the concentration of animal requirement. However, this Cu concentration was still within the maximum tolerable concentration (100.0 mg kg^-1^) for animals (Olson, 2007). The aboveground Mn concentration ranged from 75.3 mg kg^-1^ to 117.4 mg kg^-1^ under different N treatments in our study (**Figure 2B**), which meets the nutrient requirements of animals (25–300 mg kg^-1^) (Healy et al., 2016). The Na aboveground concentration had the maximum value (115.9 mg kg^-1^) in the N2 treatment, whereas the range varied from 61.1 mg kg^-1^ to 115.9 mg kg^-1^ (**Figure 2B**). These results indicated that the aboveground biomass Na concentration was not sufficient to meet animal requirements in the range from 600 mg kg^-1^to 800 mg kg^-1^ (National Research Council, 2001), especially when the higher requirements (1000 mg kg^-1^) are considered (National Research Council, 2001; Taiz and Zeiger, 2010). We consider the concentration changes of Cu, Mn and Na in the aboveground tissues of *B. ischaemum* after N addition needed to meet animal requirements. We then conclude that the concentration of fertilizer was suitable in N2.

Roots are the primary location for macro- and micronutrient uptake; therefore, the concentrations of elements are usually much higher in roots than in leaves (Vymazal et al., 2007). The macro- and micronutrient concentration ratios of above- to belowground tissues in *B. ischaemum* were < 1 in this study. Cu, Zn, Mn, and Ca concentration ratios of above- to belowground tissues varied and were significantly affected by N addition, which supported our hypothesis. A previous study indicated that the average values of the concentration ratio of the above- to belowground levels were 0.45 (Cu) and 0.57 (Zn) (Vymazal et al., 2007), which were slightly lower than the values of 0.50 (Cu) and 0.73 (Zn) in our study (**Table 2**).

### Effects of N Addition on Macro- and Micronutrient Storage in Above- and Belowground Biomass

Most of the macro- and micronutrient storage in the aboveground tissues significantly increased at low N addition, whereas the storage of Mn significantly decreased at the highest N addition level. The macro- and micronutrient storage in the belowground tissues significantly increased with N addition. However, the K, Ca, Mn, and Na storage significantly decreased with the highest N addition level (**Figures 3A–D**). These results are consistent with our hypotheses. Correlation analysis showed that the macro- and micronutrient storage in the aboveground and belowground tissues had a significant correlation with the above- and belowground biomasses under N addition treatment (**Table 3**). This finding suggested that the change in the macro- and micronutrient storage in *B. ischaemum* was mainly due to the change in biomasses after N addition. Furthermore, correlation analysis showed that the aboveground Cu and the belowground K concentrations had a significant relationship with macro- and micronutrient storage in the above- and belowground tissues, respectively (**Table 3**). As previously reported, the adsorption of macro- and micronutrients by the roots is affected by the concentrations of other elements in soil (Taiz and Zeiger, 2010). Thus, the adsorption of macro- and micronutrients by *B. ischaemum* may be affected by aboveground Cu and belowground K concentrations. N addition could significantly affect the distribution pattern of macro- and micronutrient storage in *B. ischaemum*, except for Fe and Mg. The storage ratios of above- to belowground Cu, Mn, Zn, Na, K, and Ca significantly increased under higher N addition, which were consistent with our hypothesis and previous study (Sinkhorn, 2007).

## Conclusion

The macro- and micronutrients in *B. ischaemum* and its surrounding soils varied significantly across N addition treatments. Soil Cu concentration was the most significantly changed element among the macro- and micronutrients in soil. Soil TN concentration was the most significant variable among the main soil nutrient factors. The concentration and storage of macro- and micronutrients in *B. ischaemum* above- and belowground biomass exhibited maximum concentration and storage when the N application was 5 g N m^-2^ y^-1^. Furthermore, *B. ischaemum* could allocate and accumulate increased macro- and micronutrients in the aboveground tissues and achieve high total storage when the amount of N addition was 5 g N m^-2^ y^-1^. Given that *B. ischaemum* is a forage plant and must meet animal requirements, we conclude that the best N addition level should be 5 g N m^-2^ y^-1^. These findings will provide a scientific reference for grassland management on Loess Plateau in China, given the need for macro- and micronutrients in plants and grazing livestock. This study clearly demonstrated the effects of N addition on the macro- and micronutrients in *B. ischaemum* and its surrounding soils. However, this was a controlled experiment at the institute. Therefore, the results will be affected by changes in the field environment to a certain extent. As we have discussed, the effects of N application on the macro- and micronutrients in different plant species were significantly different. Thus, changes in the macro- and micronutrients in other plant species remain uncertain. The effects of N addition on the macro- and micronutrients in *B. ischaemum* and other plant species in the field must be further studied.

## Author Contributions

GL, GW, and SX provided research ideas and designed the experiments. They were also responsible for the revision of the paper. CL, HL, and JZ participated in the implementation of the experiment, sample collection, and laboratory analysis. ZA wrote the paper and participated in the implementation of the experiment, sample collection, laboratory analysis, and data analysis.

## Conflict of Interest Statement

The authors declare that the research was conducted in the absence of any commercial or financial relationships that could be construed as a potential conflict of interest.
